# Sustained weight reduction following 12 weeks of intermittent fasting intervention in people with insulin‐treated type 2 diabetes—Two‐year follow‐up of the randomised controlled InterFast‐2 trial

**DOI:** 10.1111/dom.16158

**Published:** 2025-01-02

**Authors:** Kehkishan Azhar, Anna Ramirez‐Obermayer, Caren Sourij, Lisa Knoll, Eva Andritz, Harald Kojzar, Alexander Müller, Othmar Moser, Norbert J. Tripolt, Peter N. Pferschy, Faisal Aziz, Harald Sourij

**Affiliations:** ^1^ Interdisciplinary Metabolic Medicine Trials Unit, Division of Endocrinology and Diabetology Medical University of Graz Graz Austria; ^2^ Department of Psychiatry, Psychosomatics and Psychotherapeutic Medicine, Division of Psychiatry and Psychotherapeutic Medicine Medical University of Graz Graz Austria; ^3^ Department of Psychiatry, Psychosomatics and Psychotherapeutic Medicine, Division of Medical Psychology, Psychosomatics and Psychotherapy Medical University of Graz Graz Austria; ^4^ Department of Cardiology Medical University of Graz Graz Austria; ^5^ Division of Exercise Physiology and Metabolism, Bayreuth Centre of Sports Science University of Bayreuth Bayreuth Germany

**Keywords:** insulin therapy, intermittent fasting, type 2 diabetes, weight loss

## 1 BACKGROUND

Various therapeutic dietary interventions have proven beneficial in controlling cardiometabolic risk factors in type 2 diabetes (T2D).^1^, ^2^ Intermittent fasting (IF) which includes approaches like time‐restricted eating and alternate‐day fasting is an emerging nutritional strategy.^3^ The InterFast‐2 study demonstrated the efficacy and safety of an IF regimen in people with insulin‐treated T2D.^4^ However, the intervention lasted only 12 weeks, and long‐term efficacy and safety data on IF are missing in this population.^5^, ^6^ Hence, we conducted a follow‐up investigation of InterFast‐2 trial participants with the aim to assess the impact of 12‐week IF on body weight and glycaemic control of the participants over 2 years.

## 2 METHODS

InterFast‐2 was a single‐centre randomised controlled trial conducted at the University Hospital Graz, Graz, Austria from September 2019 to January 2022 to determine the efficacy and safety of IF in improving glycaemic and weight control in individuals with insulin‐treated T2D compared to a control group. It enrolled 46 individuals aged 18–75 years with HbA_1c_ ≥ 53 mmol/mol (≥7.0%), and a daily insulin dose ≥0.3 IU. The participants were randomly assigned to either 12 weeks of IF group (*n* = 22) or a control group (*n* = 24). The IF group followed a 3‐day per week IF regimen, during which calorie intake was restricted to a maximum of 500 kcal prior to noon. Additionally, participants were given an insulin dose adjustment protocol for fasting days to minimise the risk of hypoglycaemia.^7^ However, participants in the control group had no dietary restrictions. The co‐primary outcomes of the InterFast‐2 study were changes in HbA_1c_ levels and the proportion of individuals achieving a composite end‐point of weight reduction (≥2%), insulin dose reduction (≥10%) and HbA_1c_ reduction (≥3 mmol/mol) from baseline to 12 weeks. After 12 weeks, the IF group exhibited a significant decline in HbA_1c_ levels (*p* < 0.05) and a higher achievement of the composite outcome (*p* < 0.05) compared to the control group.

After 12‐week intervention, the participants were not given any specific instructions about following or stopping the IF. However, they were advised to follow the recommended nutritional guidelines. All participants were informed about the follow‐up visit at the end of the main trial, and informed consent was obtained for voluntary participation in the 2‐year follow‐up study. There were no telephone calls or on‐site visits prior to the follow‐up. Anthropometric, laboratory, clinical and medication data were collected during follow‐up. Additionally, the IF group participants were asked whether they followed IF from 12 weeks onwards, and a self‐reported account of IF was recorded. The outcomes of the follow‐up study were changes in HbA_1c_ levels, body weight and total daily insulin dose from baseline to 2 years as well as the proportion of participants achieving the composite end‐point after 2 years.

The InterFast‐2 trial and follow‐up study were approved by the Ethics Committee of the Medical University of Graz, Graz, Austria (EK 30‐350 ex 17/18) and adhered to the principles of Good Clinical Practice and the Declaration of Helsinki.

Continuous outcomes were presented as mean ± SD or median (interquartile range [IQR]) for continuous variables. The mean changes in body weight and HbA_1c_ levels from baseline to 2 years and from 12 weeks to 2 years were compared between the IF and control groups using linear regression, while the median change in total daily insulin dose between groups was compared using quantile regression. Both models were adjusted for baseline values of the outcomes. The achievement rate of combined outcome at 2 years was compared between groups using the Fisher's exact test. Additionally, within the IF group, the outcomes were compared between those who continued IF and those who did not. The median changes in the outcome parameters from baseline to 2 years were compared using quantile regression and adjusted for the baseline values of the outcomes.

## 3 RESULTS

After 2 years, 39 participants (89%) returned for a follow‐up visit, including 17 from the IF group and 22 from the control group. The mean age of the participants was 64.4 ± 7.3 years, T2D duration was 22.6 ± 9.3 years, weight was 97.8 ± 15.2 kg, HbA_1c_ level was 68.3 ± 15.0 mmol/mol (8.4 ± 3.5%) and median daily insulin dose was 50 (36; 72) IU.

From baseline to 2 years, the IF group showed a significant mean weight reduction of 4.2 ± 4.3 kg compared to 0.4 ± 5.4 kg in the control group, from baseline to 2 years (*p* = 0.021). The mean HbA_1c_ level decreased by 2.1 ± 11.7 mmol/mol in the IF group over time, whereas it increased by 2.3 ± 12.0 mmol/mol in the control group without a statistical significance (*p* = 0.317). After 2 years, four (23.5%) participants achieved the combined end‐point in the IF group, compared to three (13.6%) participants in the control group (*p* = 0.677). The median daily insulin dose decreased by −5 (−14; 3) IU in the IF group compared to 4 (−2; 10) IU increase in the control group (*p* = 0.301) (see Table 1).

**TABLE 1:** Comparison of body weight, HbA1c and total daily insulin dose from baseline to 2 years and 12 weeks to 2 years between intermittent fasting and control groups in InterFast‐2 participants (*N* = 39).

Outcomes	Baseline		12 weeks		2 years		Change from baseline to 2 years		*p* value	Change from 12 weeks to 2 years		*p*‐value
	IF	Control	IF	Control	IF	Control	IF	Control		IF	Control	
Weight (kg) Mean ± SD	95.0 (±14.1)	103.6 (±13.3)	90.9 (±14.2)	104.0 (±13.6)	90.8 (±13.5)	103.2 (±14.4)	−4.2 (±4.3)	−0.4 (±5.4)	0.021	−0.1 (± 4.5)	−0.8 (±5.0)	0.962
HbA_1c_ (mmol/mol) Mean ± SD	69.3 (±13.9)	66.9 (±10.3)	61.5 (±12.2)	66.0 (±10.0)	67.2 (±17.3)	69.1 (±13.2)	−2.1 (±11.7)	2.3 (±12.0)	0.317	5.8 (± 15.7)	3.1 (± 10.9)	0.746
HbA_1c_ (%) Mean ± SD	8.5 (±1.3)	8.3 (±0.9)	7.8 (±1.1)	8.2 (±0.9)	8.3 (±1.6)	8.5 (±1.2)	−0.2 (±1.1)	0.2 (±1.1)	0.317	0.5 (±1.4)	0.3 (±1.0)	0.746
TDI dose (IU) Median (IQR)	50 (36;60)	47 (34;76)	42 (30;52)	47.5 (37;80)	45 (31;60)	57 (44;80)	−5 (−14;3)	4 (−2;10)	0.301*	1 (−1;12)	1 (−10;8)	0.547*

*Note*: *p*‐values of change from baseline to 2 years and 12 weeks to 2 years are reported for linear regression and adjusted for baseline values of the parameters.

Abbreviations: HbA_1c_, glycated haemoglobin; IF, intermittent fasting; IQR, interquartile range; SD, standard deviation; TDI, total daily insulin dose.

* *p*‐value for change in total daily insulin dose from baseline to 2 years and 12 weeks to 2 years is reported for quantile regression and adjusted for baseline values of the parameter.

From 12 weeks to 2 years, an insignificant decrease in mean body weight was observed in both study groups (*p* = 0.962). The mean HbA_1c_ levels increased in both groups, with a greater increase among the IF group participants (*p* = 0.746), without statistical significance. Similarly, there was an insignificant increase in the median total daily insulin dose in both groups (*p* = 0.547). The details are presented in Table 1.

From baseline to 2 years, among the 17 participants randomised to IF group, seven (41.2%) discontinued IF after 12 weeks, while 10 (58.8%) continued IF, at least infrequently, for up to 2 years. Those who continued IF, experienced a higher median weight loss of 4.5 kg (−8.4; −1.4 kg) versus 3.1 kg (−4.1; 0.1 kg) among those who discontinued IF, though the difference was not statistically significant (*p* = 0.221). Also, those who continued IF showed median reductions in HbA_1c_ and daily insulin dose of −4.5 (−8; −2) mmol/mol and −5.5 (−16; −3) IU, respectively, compared to an increase in both parameters among those who discontinued IF over 2 years. However, these differences were not statistically significant (Table 2). No severe hypoglycaemic events (requiring third‐party assistance or hospitalisation) were reported by any of the study participants over the 2 years of follow up.

**TABLE 2:** Comparison of body weight, HbA_1c_ and total daily insulin dose from baseline to 2 years between participants who continued intermittent fasting (IF) at least infrequently and those who did not continue IF at all during the follow‐up in the InterFast‐2 participants allocated to IF group (*N* = 17).

Outcomes	Baseline median (IQR)		2 years median (IQR)		Change from baseline to 2 years median (IQR)		*p*‐value
	IF	No IF	IF	No IF	IF	No IF	
Weight (kg)	94.2 (88.9; 107.4)	84.1 (83.1; 94.9)	89.0 (84.0; 99.0)	83.0 (80.0; 95.0)	−4.5 (−8.4; −1.4)	−3.1 (−4.1; 0.1)	0.221
HbA_1c_ (mmol/mol)	63.0 (57.0; 76.0)	65.0 (63.0; 98.0)	56.0 (54.0; 66.0)	72.0 (66.0; 82.0)	−4.5 (−8; −2)	8 (−6; 13)	0.119
HbA_1c_ (%)	7.9 (7.4; 9.1)	8.2 (7.9; 11.1)	7.3 (7.1; 8.2)	8.7 (8.2; 9.6)	−0.4 (−0.7; −0.2)	0.7 (−0.5; 1.2)	0.119
TDI dose (IU)	45.0 (36.0; 56.0)	57.0 (44.0; 82.0)	38.0 (25.0; 51.0)	51.0 (43.0; 112.0)	−5.5 (−16; −3)	3 (−7; 30)	0.542

*Note*: *p*‐values of change from baseline to 2 years are reported for quantile regression and adjusted for baseline values of the parameters.

Abbreviations: HbA_1c_, glycated haemoglobin; IF, intermittent fasting; IQR, interquartile range; TDI, total daily insulin dose.

## 4 CONCLUSIONS

In our analysis, a 12‐week IF intervention led to a significant and sustainable weight loss over 2 years compared to the control group. Even though the frequency of IF and caloric restriction was not maintained by those orignially randomised to IF, the average body weight was still significantly lower than the baseline average body weight in the IF group compared to the control group.

However, from 12 weeks to 2 years, a small and statistically insignificant weight loss was observed in both the groups. This could be due to a lower number of participants following IF with variable frequency and caloric restriction, as they were not advised or tracked for adherence to the protocol after the end of the 12‐week intervention. A recent systematic review and meta‐analysis of interventional studies has reported a positive role of IF in reducing weight in individuals with T2D.^8^ Similarly, a prospective analysis of a randomised controlled trial reported a sustained weight loss of approximately 4 kg among the participants following intermittent energy restriction for 2 years (500–600 kcal diet for 2 days/week).^9^ These consistent findings across studies in heterogeneous T2D populations endorse the role of IF even as a short‐term intervention in sustainably reducing body weight in people with T2D.

Despite an increase in the mean HbA_1c_ level in the IF group from 12‐weeks to 2 years, it remained lower than its baseline level. In contrast, the mean HbA_1c_ level increased slightly from baseline to 2 years and from 12 weeks to 2 years in the control group. A similar pattern was observed for total daily insulin dose. Additionally, the combined end‐point was achieved by more participants in the IF group than in the control group, albeit with a statistically insignificant difference between the groups, which could be due to the inadequate study power at the 2‐year follow‐up. Moreover, comparing the outcomes within the IF group, participants who continued IF experienced greater reductions in weight, HbA_1c_ levels and total daily insulin dose than those who discontinued IF after 12 weeks. Although these results were statistically insignificant, even this magnitude of reduction in HbA_1c_ levels could still be clinically beneficial in the management of T2D and provides a basis for future research in this area.

Evidence supports that sustained glycaemic control significantly reduces the odds of developing T2D‐related cardiovascular and renal complications.^10^ Furthermore, a meta‐analysis of randomised controlled trials has shown that dietary interventions are more effective than standard care in improving glycaemic control, particularly in cases of weight loss.^11^ However, we did not observe any significant improvement in glycaemic control at the 2‐year follow‐up, which could be due to the lack of monitoring and contact with participants after the 12‐week intervention. Additionally, almost 40% of the IF group participants discontinued IF, limiting the data on feasibility of sustained adherence to IF over a longer period of time. Furthermore, it highlights the challenges of long‐term dietary interventions and demands further research to evaluate continuous IF adherence, particularly among insulin‐treated T2D individuals. Nevertheless, our results support the potential of annual 12‐week IF bouts as a reasonable dietary strategy for T2D management. Given that no severe hypoglycaemic events (those requiring third‐party assistance or hospitalisation) were observed among participants who continued IF for 2 years, this approach can be considered safe for implementation across diverse T2D populations. However, it is crucial to emphasise that IF requires constant medical supervision, vigilant self‐monitoring and treatment adjustments for effective implementation.

A limitation of our study is the small sample size; however, 89% of the participants from the main trial participated in the follow‐up study. Hence, the response rate was adequate. Moreover, we do not have a continuous record of IF frequency and degree of caloric restriction which was performed from the end of the 12‐week intervention until the 2‐year follow‐up period and had to rely on patient reporting.

Our results suggest that IF is an effective strategy for achieving sustained weight loss, and if followed appropriately under the supervision and guidance of healthcare providers, it could aid in achieving glycaemic control in various T2D populations. However, more prospective studies, potentially including annual IF interventions, are recommended to evaluate the long‐term efficacy and safety of various caloric‐restriction approaches in diverse diabetes cohorts.

## AUTHOR CONTRIBUTIONS

K.A. wrote the final manuscript. A.Ob., C.S., L.K., E.A., H.K., A.M., P.N.P and N.J.T contributed to the collection and interpretation of data. K.A. and F.Az. contributed to the statistical analysis. N.J.T. and H.S. contributed to acquiring ethical approval for the trial. H.S. conceived the trial. O.M and all authors reviewed and contributed to the final manuscript. H.S is the guarantor of this work and, as such, had full access to all data in the study and takes responsibility for the integrity of the data and the accuracy of the data analysis.

## FUNDING INFORMATION

This research was funded in whole or in part by the Austrian Science Fund (FWF) [10.55776/PIN8074224] grants KLI 851‐B and KLI‐1076 to Harald Sourij.

Clinical trial registration number is DRKS00018070, Deutschen Register Klinischer Studien (DRKS).

## CONFLICT OF INTEREST STATEMENT

All authors declare no conflict of interest relevant to this article.

### PEER REVIEW

The peer review history for this article is available at https://www.webofscience.com/api/gateway/wos/peer‐review/10.1111/dom.16158.

## Data Availability

The dataset analysed in this study is available from the corresponding author upon reasonable request.
